# Comparison of complementary and alternative medicine with conventional mind–body therapies for chronic back pain: protocol for the Mind–body Approaches to Pain (MAP) randomized controlled trial

**DOI:** 10.1186/1745-6215-15-211

**Published:** 2014-06-07

**Authors:** Daniel C Cherkin, Karen J Sherman, Benjamin H Balderson, Judith A Turner, Andrea J Cook, Brenda Stoelb, Patricia M Herman, Richard A Deyo, Rene J Hawkes

**Affiliations:** 1 Group Health Research Institute, 1730 Minor Avenue, Suite 1600, Seattle, WA 98101, USA; 2 Departments of Family Medicine and Health Services, University of Washington, Box 357660, Seattle, WA 98195, USA; 3 Department of Epidemiology, University of Washington, Box 357236, Seattle, WA 98195, USA; 4 Departments of Psychiatry and Behavioral Sciences and Rehabilitation Medicine, University of Washington, Box 356560, Seattle, WA 98195, USA; 5 Department of Biostatistics, University of Washington, Box 357232, Seattle, WA 98195, USA; 6 RAND Corporation, 1776 Main Street, Santa Monica, CA, USA; 7 Departments of Family Medicine, Internal Medicine, Public Health and Preventive Medicine, and the Center for Research in Occupational and Environmental Toxicology, Oregon Health and Science University, 3181 SW Sam Jackson Park Road, Portland, OR 97239, USA

**Keywords:** Back pain, Cognitive-behavioral therapy, Mindfulness meditation

## Abstract

**Background:**

The self-reported health and functional status of persons with back pain in the United States have declined in recent years, despite greatly increased medical expenditures due to this problem. Although patient psychosocial factors such as pain-related beliefs, thoughts and coping behaviors have been demonstrated to affect how well patients respond to treatments for back pain, few patients receive treatments that address these factors. Cognitive-behavioral therapy (CBT), which addresses psychosocial factors, has been found to be effective for back pain, but access to qualified therapists is limited. Another treatment option with potential for addressing psychosocial issues, mindfulness-based stress reduction (MBSR), is increasingly available. MBSR has been found to be helpful for various mental and physical conditions, but it has not been well-studied for application with chronic back pain patients. In this trial, we will seek to determine whether MBSR is an effective and cost-effective treatment option for persons with chronic back pain, compare its effectiveness and cost-effectiveness compared with CBT and explore the psychosocial variables that may mediate the effects of MBSR and CBT on patient outcomes.

**Methods/Design:**

In this trial, we will randomize 397 adults with nonspecific chronic back pain to CBT, MBSR or usual care arms (99 per group). Both interventions will consist of eight weekly 2-hour group sessions supplemented by home practice. The MBSR protocol also includes an optional 6-hour retreat. Interviewers masked to treatment assignments will assess outcomes 5, 10, 26 and 52 weeks postrandomization. The primary outcomes will be pain-related functional limitations (based on the Roland Disability Questionnaire) and symptom bothersomeness (rated on a 0 to 10 numerical rating scale) at 26 weeks.

**Discussion:**

If MBSR is found to be an effective and cost-effective treatment option for patients with chronic back pain, it will become a valuable addition to the limited treatment options available to patients with significant psychosocial contributors to their pain.

**Trial registration:**

Clinicaltrials.gov Identifier: NCT01467843.

## Background

Identifying cost-effective treatments for chronic low back pain (CLBP) remains a challenge for clinicians, researchers, payers and patients. About $26 billion is spent annually in the United States in direct costs of medical care for back pain [1]. In 2002, the estimated costs of lost worker productivity due to back pain were $19.8 billion [2]. Despite numerous options for evaluating and treating back pain, as well as the greatly increased medical care resources devoted to this problem, the health and functional status of persons with back pain in the United States has deteriorated [3]. Furthermore, both providers and patients are dissatisfied with the status quo [4-6] and continue to search for better treatment options.

There is substantial evidence that patient psychosocial factors, such as pain-related beliefs, thoughts and coping behaviors, can have a significant impact on the experience of pain and its effects on functioning [7]. This evidence highlights the potential value of treatments for back pain that address both the mind and the body. In fact, four of the eight nonpharmacologic treatments recommended by the American College of Physicians and the American Pain Society guidelines for persistent back pain include “mind–body” components [8]. One of these treatments, cognitive-behavioral therapy (CBT), includes mind–body components such as relaxation training and has been found to be effective for a variety of chronic pain problems, including back pain [9-13]. CBT has become the most widely applied psychosocial treatment for patients with chronic back pain. Another mind–body therapy, mindfulness-based stress reduction (MBSR) [14,15], focuses on teaching techniques to increase mindfulness. MBSR and related mindfulness-based interventions have been found to be helpful for a broad range of mental and physical health conditions, including chronic pain [14-19], but they have not been well-studied for chronic back pain [20-24]. Only a few small pilot trials have evaluated the effectiveness of MBSR for back pain [25,26] and all reported improvements in pain intensity [27] or patients’ acceptance of pain [28,29].

Further research on the comparative effectiveness and cost-effectiveness of mind–body therapies should be a priority in back pain research for the following reasons: (1) the large personal and societal impact of chronic back pain, (2) the modest effectiveness of current treatments, (3) the positive results of the few trials in which researchers have evaluated mind–body therapies for back pain and (4) the growing popularity and safety, as well as the relatively low cost, of mind–body therapies. To help fill this knowledge gap, we are conducting a randomized trial to evaluate the effectiveness, comparative effectiveness and cost-effectiveness of MBSR and group CBT, compared with usual medical care only, for patients with chronic back pain.

### Specific aims

Our specific aims and their corresponding hypotheses are outlined below.

1. To determine whether MBSR is an effective adjunct to usual medical care for persons with CLBP

*Hypothesis 1*: Individuals randomized to the MBSR course will show greater short-term (8 and 26 weeks) and long-term (52 weeks) improvement in pain-related activity limitations, pain bothersomeness and other health-related outcomes than those randomized to continued usual care alone.

2. To compare the effectiveness of MBSR and group CBT in decreasing back pain–related activity limitations and pain bothersomeness

*Hypothesis 2*: MBSR will be more effective than group CBT in decreasing pain-related activity limitations and pain bothersomeness in both the short term and long term. The rationale for this hypothesis is based on (1) the modest effectiveness of CBT for chronic back pain found in past studies, (2) the positive results of the limited initial research evaluating MBSR for chronic back pain and (3) growing evidence that an integral part of MBSR training (but not CBT training)—yoga—is effective for chronic back pain.

3. To identify the mediators of any observed effects of MBSR and group CBT on pain-related activity limitations and pain bothersomeness

*Hypothesis 3a*: The effects of MBSR on activity limitations and pain bothersomeness will be mediated by increases in mindfulness and acceptance of pain.

*Hypothesis 3b*: The effects of CBT on activity limitations and pain bothersomeness will be mediated by changes in pain-related cognition (decreases in catastrophizing, beliefs that one is disabled by pain and beliefs that pain signals harm, as well as increases in perceived control over pain and self-efficacy for managing pain) and changes in coping behaviors (increased use of relaxation, task persistence and coping self-statements and decreased use of rest).

4. To compare the cost-effectiveness of MBSR and group CBT as adjuncts to usual care for persons with chronic back pain

*Hypothesis 4*: Both MBSR and group CBT will be cost-effective adjuncts to usual care.

We will also explore whether certain patient characteristics predict or moderate treatment effects. For example, we will explore whether patients with higher levels of depression are less likely to improve with both CBT and MBSR or whether such patients are more likely to benefit from CBT than from MBSR (that is, whether depression level is a moderator of treatment effects).

## Methods/Design

### Overview

**Figure 1:**
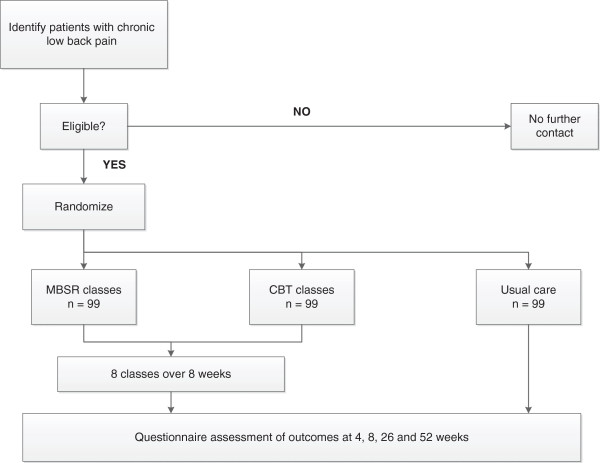
**Flowchart of the trial protocol.** CBT, Cognitive-behavioral therapy; MBSR, Mindfulness-based stress reduction.

We are conducting a randomized clinical trial in which individuals with CLBP are randomly assigned to group CBT, a group MBSR course or usual care alone (Figure 1). Participants will be followed for 52 weeks after randomization. Telephone interviewers masked to participants’ treatment assignments will assess outcomes 4, 8, 26 and 52 weeks postrandomization. The primary outcomes we will assess are pain-related activity limitations and pain bothersomeness. Participants will be informed that the study researchers are comparing “two different widely used pain self-management programs that have been found helpful for reducing pain and making it easier to carry out daily activities”.

The protocol for this trial has been approved by the Human Subjects Review Committee of the Group Health Cooperative (250681-22). All participants will be required to give their informed consent before enrollment in this study.

### Study sample and setting

The primary source of participants for this trial will be the Group Health Cooperative (GHC), a group-model, not-for-profit health-care organization that serves over 600,000 enrollees through its own primary care facilities in Washington state. As needed to achieve recruitment goals, direct mailings will be sent to persons 20 to 70 years of age living in the areas served by the GHC.

### Inclusion and exclusion criteria

We are recruiting individuals from 20 to 70 years of age whose back pain has persisted for at least 3 months. The inclusion and exclusion criteria were developed to maximize the enrollment of appropriate patients while screening out patients who have low back pain of a specific nature (for example, spinal stenosis) or a complicated nature or who would have difficulty completing the study measures or interventions (for example, psychosis). Reasons for exclusion of GHC members were identified on the basis of (1) automated data recorded (using the *International Classification of Diseases*, *Ninth Revision* coding system), during all visits over the course of the previous year and (2) eligibility interviews conducted by telephone. For non-GHC members, reasons for exclusion were identified on the basis of telephone interviews. Tables 1 and 2 list the inclusion and exclusion criteria, respectively, as well as the rationale for each criterion and the information sources.

**Table 1:** Inclusion criteria

**Inclusion criteria**	**Rationales**	**Sources**
Primary sources		A, TI
All GHC members with at least one primary care visit for back pain within the previous 3 to 15 months	Initial targeting of members with back pain visits in recent past is an efficient method of identifying persons with chronic back pain.	
Random sample of GHC members without visits for back pain within the previous 3 to 15 months	Emphasis placed on recruitment of GHC members to obtain complete treatment utilization and cost data from GHC medical records	
GHC members responding to an ad in the GHC magazine		
Secondary sources		A, TI
Random samples of nonmembers of GHC living in Washington state	Nonmembers of GHC included to ensure that recruitment goals are met.	
Age range 20 to 70 years	Chronic back pain in children is a result of causes different from those we will study.	
	Adults have a higher risk of undiagnosed serious conditions that cause back pain.	
Nonspecific, uncomplicated back pain as defined by ICD-9 codes (for primary source only: GHC members with primary care visits for back pain in the previous 3 to 15 months)	These codes are consistent with low back pain that is nonspecific and uncomplicated.	A
724.2 Lumbago		
724.5 Backache, unspecified		
724.8 Other symptoms referable to back		
846.0-9 Sprains and strains, sacroiliac		
847.2 Sprains and strains, lumbar		
847.3 Sprains and strains, sacral		
847.9 Sprains and strains, unspecified site of the back		

GHC, Group Health Cooperative; ICD-9, *International Classification of Diseases*, *Ninth Revision*. A, Automated data gathered from visits; TI, Telephone interview.

**Table 2:** **Exclusion criteria**^**a**^

**Exclusion criteria**	**Rationales**	**Sources**
Patient claims visit was not for low back pain	Study restricted to low back pain	TI
Low back pain has lasted <3 months	Low back pain not chronic	TI
Self-rated bothersomeness of pain rating of <4 (on 0 to 10 scale) and pain interference with activities rating <3 (on 0 to 10 scale) during previous week	Back pain too mild to detect improvement	TI
Abdominal aneurysm	Back pain due to or possibly result of specific disease or condition	A
Cancer in previous 5 years, other than basal cell or squamous cell cancer of the skin		A, TI
Discitis		A
Degenerative disc disease		A
Fracture of vertebra		A, TI
Infectious cause of back pain		TI
Pregnancy		TI
Scoliosis, severe or progressive		A, TI
Spinal stenosis		A, TI
Spondylolisthesis		A, TI
Back surgery within previous 2 years	Back problem is complicated by medical or medicolegal issues	TI
Seeking or receiving compensation for back pain or involved in litigation related to back pain		
Blindness	Condition might make it difficult to attend the classes or practice at home	A
Deafness		A
No way to play home practice audio recordings at home		TI
Paralysis		A
Psychoses, major		A, TI
Schedules do not permit participation in classes or home practice (including planning to move out of town)		TI
Vision problems, severe		TI
Hearing problems, severe		TI
Lack of transportation		TI
Fibromyalgia	Condition or circumstance might confound treatment effects or interpretation of data	TI
Rheumatoid arthritis and/or anklyosing spondylitis		A, TI
Other disabling chronic conditions (for example, disabling heart or lung disease, diabetic neuropathy, receiving treatment for hepatitis)		TI
Planning on seeing a specialist for back pain (for example, surgeon, neurologist, rheumatologist)		TI
Dementia	Condition would make it difficult to provide fully informed consent	A
Unable to read or speak English		TI
Currently taking a mind–body class for back pain or class taken within previous 1 year	Possible bias due to current or recent interventions	TI

^a^A, Automated data gathered from visits; TI, Telephone interview.

In addition, we require that participants be willing and able to attend the CBT or MBSR classes during the 8-week intervention period if assigned to one of those treatments, and to respond to the four follow-up questionnaires so that we can assess outcomes.

### Recruitment procedures

Because the study intervention involves classes, we are recruiting participants in ten cohorts consisting of up to forty-five individuals each. We are recruiting participants from three main sources: (1) GHC members who have made visits to their primary care providers for low back pain and whose pain has persisted for at least 3 months, (2) GHC members who have not made a visit to their primary care provider for back pain but who are between the ages of 20 and 70 years and who respond to our nontargeted GHC mailing or our ad in GHC’s twice-yearly magazine and (3) community residents between the ages of 20 and 70 years who respond to a direct mail recruitment postcard.

For the targeted GHC population, a programmer will use GHC’s administrative and clinical electronic databases to identify potentially eligible members with a visit in the previous 3 to 15 months to a provider that resulted in a diagnosis consistent with nonspecific low back pain. These GHC members are mailed a letter and consent checklist that explains the study and eligibility requirements. Members interested in participating sign and return a statement indicating their willingness to be contacted. A research specialist then calls the potential participant to ask questions; determine eligibility; clarify risks, benefits and expected commitment to the study; and request informed consent. After informed consent has been obtained from the individual, the baseline telephone assessment is conducted.

For the nontargeted GHC population (that is, GHC members without visits with back pain diagnoses received within the previous 3 to 15 months but who could possibly have low back pain), a programmer uses administrative and clinical electronic databases to identify potentially eligible members who were not included in the targeted sample described in the preceding paragraph. This population also includes GHC members who respond to an ad in the GHC magazine. The same methods used for the targeted population are then used to contact and screen the potential participants, obtain their informed consent and collect baseline data.

With regard to community residents, we have purchased lists of the names and addresses of a randomly selected sample of people living within our recruitment area who are between 20 and 70 years of age. The people on the list are sent direct mail postcards describing the study including information regarding how to contact study staff if interested in participating. Once an interested person has contacted the research team the same process detailed above is followed.

To ensure that all initially screened study participants remain eligible at the time the classes begin, those who consent more than 14 days prior to the start of the intervention classes will be recontacted approximately 0 to 14 days prior to the first class to reconfirm their eligibility. The primary concern is to exclude persons who no longer have at least moderate baseline ratings of pain bothersomeness and pain-related interference with activities. Those individuals who remain eligible and give their final informed consent will be administered the baseline questionnaire.

### Randomization

After completing the baseline assessment, participants will be randomized in equal proportions to the MBSR, CBT or usual care group. Those randomized to the MBSR or CBT group will not be informed of their type of treatment until they arrive at the first classes, which will occur simultaneously in the same building. The intervention group will be assigned on the basis of a computer-generated sequence of random numbers using a program which ensures that allocation cannot be changed after randomization. To ensure balance on a key baseline prognostic factor, randomization will be stratified based on our primary outcome measurement instrument: the modified version of the Roland Disability Questionnaire (RDQ) [30,31]. We will stratify participants into two activity limitations groups: moderate (RDQ score ≤12 on a 0 to 23 scale) and high (RDQ scores ≥13). Participants will be randomized within these strata in blocks of varying size (three, six or nine) to ensure a balanced but unpredictable assignment of participants. During recruitment, the study biostatistician will receive aggregated counts of participants randomized to each group to assure that the preprogrammed randomization algorithm is functioning properly.

### Study treatments

Both the group CBT and MBSR class series consist of eight weekly 2-hour sessions supplemented by home activities.

#### Mindfulness-based stress reduction

Mindfulness-based stress reduction, a 30-year-old treatment program developed by Jon Kabat-Zinn, is well-described in the literature [32-34]. The authors of a recent meta-analysis found that MBSR had moderate effect sizes for improving the physical and mental well-being of patients with a variety of health conditions [16]. Our MBSR program is closely modeled on the original one and includes eight weekly 2-hour classes (summarized in Table 3), a 6-hour retreat between weeks 6 and 7 and up to 45 minutes per day of home practice. Our MBSR protocol was adapted by a senior MBSR instructor from the 2009 MBSR instructor’s manual used at the University of Massachusetts [35]. This manual permits latitude in how instructors introduce mindfulness and its practice to participants. The handouts and home practice materials are standardized for this study.

**Table 3:** **Content of cognitive-behavioral therapy and mindfulness-based stress reduction class sessions**^**a**^

**Session**	**CBT**	**MBSR**
1	Rationale and evidence base for CBT, pain neurobiology, overview of skills, rationale for relaxation training, diaphragmatic breathing instruction, introduction to behavioral goal setting (physical activity and home practice)	Setting expectations, defining mindfulness, engaging in mindfulness exercise and movement, abdominal breathing instructions, introducing home practice
2	Behavioral goal setting, physical activity and pacing activity, pleasant event scheduling, pain flare-up plan, diaphragmatic breathing and seven-muscle-group progressive muscle relaxation (PMR) instruction	Perception and responding creatively to perceptions, engaging in yoga, body scan, discussion of developing a practice
3	Role of thoughts and feelings in pain, introduction to the CBT model, thought-monitoring record, diaphragmatic and four-muscle-group PMR instruction, setting and working toward behavioral goals	Pleasure and power of being present, engaging in yoga, walking meditation, discussing how to bring more pleasant events into our lives
4	Challenging automatic thoughts related to pain, setting and working toward behavioral goals, diaphragmatic breathing and four-muscle-group/no-tension PMR instruction	Getting unstuck from old patterns, engaging in yoga and/or movement, sitting meditation, stress (define and identify how and why we stay stuck), dealing with pain
5	Challenging automatic thoughts and generating alternate thoughts, distraction techniques, brief body scan relaxation instruction, setting and working toward behavioral goals	Reacting and responding differently to stress, engaging in yoga, participating in guided meditation, establishing coping strategies (living with difficulties)
6	Alternate thoughts, thought stopping, behavioral activation, mini-relaxation instruction, setting and working toward behavioral goals	Learning about communication patterns, engaging in yoga and/or meditation, styles of communicating with others (effective and ineffective)
7	Rehearsing pain coping skills, identifying and challenging negative thoughts, setting and working toward behavioral goals, mini-relaxation instruction, sleep tips	Integrating learning from techniques, yoga and/or meditation, practical ways to bring mindfulness into daily life
8	Maintenance of coping skills, relapse prevention, personal plan for the future	This is the rest of your life, review mindfulness techniques and applications, closure

^a^CBT, Cognitive-behavioral therapy; MBSR, Mindfulness-based stress reduction.

Participants will be given a packet of information during the first class that includes a course outline and instructor contact information; information about mindfulness, meditation, communication skills and effects of stress on the body, emotions and behavior; homework assignments; poems; and a bibliography. All sessions will include mindfulness exercises, and all but the first will include yoga or other forms of mindful movement. Participants will be given audio recordings of the mindfulness and yoga techniques, which will have been recorded by their own instructors. Participants will be asked to practice the techniques discussed in each class daily for up to 45 minutes throughout the intervention period and after classes end. They will also be assigned readings to complete before each class. Time will be devoted in each class to a review of challenges that participants have had in practicing what they learned in previous classes and with their homework. An optional day of practice on the Saturday between the sixth and seventh classes will be offered. This 6-hour “retreat” will be held with the participants in silence and only the instructor speaking. This will provide participants an opportunity to deepen what they have learned in class.

#### Cognitive-behavioral therapy

CBT for chronic pain is well-described in the literature and has been found to be modestly to moderately effective in improving chronic pain problems [9-13]. There is no single, standardized CBT intervention for chronic pain, although all CBT interventions are based on the assumption that both cognition and behavior influence adaptation to chronic pain and that maladaptive cognition and behavior can be identified and changed to improve patient functioning [36]. CBT emphasizes active, structured techniques to teach patients how to identify, monitor and change maladaptive thoughts, feelings and behaviors, with a focus on helping patients to acquire skills that they can apply to a variety of problems and collaboration between the patient and therapist. A variety of techniques are taught, including training in pain coping skills (for example, use of positive coping self-statements, distraction, relaxation and problem-solving). CBT also promotes setting and working toward behavioral goals.

Both individual and group formats have been used in CBT. Group CBT is often an important component of multidisciplinary pain treatment programs. We will use a group CBT format because it has been found to be efficacious [37-40], is more resource-efficient than individual therapy and provides patients with the potential benefits deriving from contact with, and support and encouragement from, others with similar experiences and problems. In addition, using group formats for both MBSR and CBT will eliminate intervention format as a possible explanation for any differences observed between the two therapies.

For this study, we developed a detailed therapist’s manual with content specific for each session, as well as a participant’s workbook containing materials for use in each session. We developed the therapist’s manual and participant’s workbooks based on existing published resources as well as on materials we have used in prior studies [39-47].

The CBT intervention (Table 3) will consist of eight weekly 2-hour sessions that will provide (1) education about the role of maladaptive automatic thoughts (for example, catastrophizing) and beliefs (for example, one’s ability to control pain, hurt equals harm) common in people with depression, anxiety and/or chronic pain and (2) instruction and practice in identifying and challenging negative thoughts, the use of thought-stopping techniques, the use of positive coping self-statements and goal-setting, relaxation techniques and coping with pain flare-ups. The intervention will also include education about activity pacing and scheduling and about relapse prevention and maintenance of gains. Participants will be given audio recordings of relaxation and imagery exercises and asked to set goals regarding their relaxation practice. During each session, participants will complete a personal action plan for activities to be completed between sessions. These plans will be used as logs for setting specific home practice goals and checking off activities completed during the week to be reviewed at the next week’s session.

#### Usual care

The usual care group will receive whatever medical care they would normally receive during the study period. To minimize possible disappointment with not being randomized to a mind–body treatment, participants in this group will receive $50 compensation.

### Class sites

The CBT and MBSR classes will be held in facilities close to concentrations of GHC members in Washington state (Bellevue, Bellingham, Olympia, Seattle, Spokane and Tacoma).

### Instructors

All MBSR instructors will have received either formal training in teaching MBSR from the Center for Mindfulness at the University of Massachusetts or equivalent training. They will themselves be practitioners of both mindfulness and a body-oriented discipline (for example, yoga), will have taught MBSR previously and will have made mindfulness a core component of their lives. The CBT intervention will be conducted by doctorate-level clinical psychologists with previous experience in providing CBT to patients with chronic pain.

### Training and monitoring of instructors

All CBT instructors will be trained in the study protocol for the CBT intervention by the study’s clinical psychologist investigators (BHB and JAT), who are very experienced in administering CBT to patients with chronic pain. BHB will supervise the CBT instructors. One of the investigators (KJS) will train the MBSR instructors in the adapted MBSR protocol and supervise them. Each instructor will attend weekly supervision sessions, which will include discussion of positive experiences, adverse events, concerns raised by the instructor or participants and protocol fidelity. Treatment fidelity checklists highlighting the essential components for each session were created for both the CBT and MBSR arms. A trained research specialist will use the fidelity checklist during live observation of every session. The research specialist will provide feedback to the supervisor to facilitate weekly supervision of the instructors. In addition, all sessions will be audio-recorded. The supervisors will listen to a random sample and requested portions of sessions and will monitor them using the fidelity checklist. Feedback will be provided to the instructors during their weekly supervision sessions. Treatment fidelity will be monitored in both intervention groups by KJS and BHB with assistance from research specialists. In addition, they will review and rate on the fidelity checklist a random sample of the recorded sessions.

### Participant retention and adherence to home practice

Participants will receive a reminder call before the first class and whenever they miss a class. They will be asked to record their daily home practice on weekly logs. Questions about their home practice during the prior week will also be included in all follow-up interviews. To maintain interviewer blinding, adherence questions will be asked after all outcome data have been recorded.

### Measures

We will assess a variety of participant baseline characteristics, including sociodemographic characteristics, back pain history and expectations of the helpfulness of the mind–body treatments for back pain (Table 4).

**Table 4:** **Baseline and follow-up measures**^**a**^

**Measures**	**Baseline**	**4 wk**	**8 wk**	**26 wk**	**52 wk**
Baseline characteristics					
Patient characteristics (age, sex, education, race, ethnicity, marital status, income, work status, number of pain sites)	x				
Back pain (pain duration, interference with activities, days of pain in previous 6 months, previous spinal injections, whether pain radiates into leg below knee)	x				
Expectations for back pain improvement in general and as result of MBSR or CBT	x				
Primary outcomes					
Back pain-related activity limitations in past week (modified RDQ)	x	x	x	x	x
Bothersomeness of back pain in past week (0 to 10 scale)	x	x	x	x	x
Secondary outcomes					
Characteristic pain intensity (GCPS) (average of pain now, worst pain, average pain)	x		x	x	x
Depression (PHQ-8)	x		x	x	x
Anxiety (GAD-2)	x		x	x	x
Medications used for back pain in past week	x		x	x	x
Exercise in past week (back pain–specific and general)	x		x	x	x
Global improvement (PGIC)			x	x	x
Program’s impact on thoughts, feelings, reactions, activities (open-ended)			x	x	x
Potential mediators					
MBSR: mindfulness (FFMQ-SF), pain acceptance (CPAQ-8)	x	x	x	x	x
CBT: pain beliefs and appraisals (PSEQ; SOPA 2-item control, disability and harm scales; PCS), pain coping strategies (CPCI activity pacing scale and 2-item relaxation scale)	x	x	x	x	x
Cost-effectiveness outcomes					
Quality of life (EQ-5D, SF-6D from the SF-12)	x	x	x	x	x
Out-of-plan visits paid for by patients since previous follow-up interview	x		x	x	x
Absenteeism, presenteeism (WPAI-CLBP)	x		x	x	x
Costs paid by GHC (payer) for back-related utilization of services (visits, tests, prescriptions) and total costs (GHC members only) (from GHC electronic medical records for year prior to trial and for follow-up year)					
Intervention-related information					
Class attendance (class records)					
Adverse experiences from classes or home practice		x	x	x	x
New serious health problems since entering study		x	x	x	x
Self-reported home practice		x	x	x	x
Perceived helpfulness of classes and home practice			x	x	x
Would recommend program to friends			x	x	x

^a^CBT, Cognitive-behavioral therapy; CPAQ-8, 8-item Chronic Pain Acceptance Questionnaire; CPCI, Chronic Pain Coping Inventory; EQ-5D, European Quality of Life (EuroQol) instrument in 5 dimensions; FFMQ, Five Facet Mindfulness Questionnaire; GAD-2, 2-item Generalized Anxiety Disorder scale; GCPS, Graded Chronic Pain Scale; MBSR, Mindfulness-based stress reduction; PCS, Pain Catastrophizing Scale; PGIC, Patient Global Impression of Change scale; PHQ-8,8 item Patient Health Questionnaire; PSEQ, Patient Self-efficacy Questionnaire; RDQ, Roland Disability Questionnaire; SF-6D, Short Form Health Survey in 6 dimensions; SF-12, 12-item Short Form Health Survey; SOPA, Survey of Pain Attitudes; WPAI-CLBP, Work Productivity and Activity Impairment Questionnaire–Chronic Low Back Pain.

We will assess a core set of outcomes for patients with spinal disorders (back-related function, pain, general health status, work disability and patient satisfaction) [48] that are consistent with the Initiative on Methods, Measurement, and Pain Assessment in Clinical Trials recommendations for clinical trials of chronic pain treatment efficacy and effectiveness [49]. We will measure both short-term outcomes (8 and 26 weeks) and long-term outcomes (52 weeks). We will also include a brief, 4-week, midtreatment assessment to permit analyses of the hypothesized mediators of the effects of MBSR and CBT on the primary outcomes. The primary study endpoint is 26 weeks. Participants will be paid $20 for each follow-up interview completed to maximize response rates.

#### Co–primary outcome measures

The co–primary outcome measures will be back-related activity limitations and back pain bothersomeness.

Back-related activity limitations will be measured with the modified RDQ, which asks whether 23 specific activities have been limited due to back pain (yes or no) [30]. We have further modified the RDQ to ask a question about the previous week rather than just “today”. The original RDQ has been found to be reliable, valid and sensitive to clinical changes [31,48,50-53], and it is appropriate for telephone administration and use with patients with moderate activity limitations [50].

Back pain bothersomeness will be measured by asking participants to rate how bothersome their back pain has been during the previous week on a 0 to 10 scale (0 = “not at all bothersome” and 10 = “extremely bothersome”). On the basis of data compiled from a similar group of GHC members with back pain, we found this bothersomeness measure to be highly correlated with a 0 to 10 measure of pain intensity (*r* = 0.8 to 0.9; unpublished data (DCC and KJS) and with measures of function and other outcome measures [54]. The validity of numerical rating scales of pain has been well-documented, and such scales have demonstrated sensitivity in detecting changes in pain after treatment [55].

We will analyze and report these co–primary outcomes in two ways. First, for our primary endpoint analyses, we will compare the percentages of participants in the three treatment groups who achieve clinically meaningful improvement (≥30% improvement from baseline) [56,57] at each time point (with 26-week follow-up being the primary endpoint). We will then examine, in a secondary outcome analysis, the adjusted mean differences between groups on these measures at the time of follow-up.

#### Secondary outcome measures

The secondary outcomes that we will measure are depressive symptoms, anxiety, pain-related activity interference, global improvement with treatment, use of medications for back pain, general health status and qualitative outcomes.

Depressive symptoms will be assessed with the Patient Health Questionnaire-8 (PHQ-8) [58]. With the exception of the elimination of a question about suicidal ideation, the PHQ-8 is identical to the PHQ-9, which has been found to be reliable, valid and responsive to change [59,60].

Anxiety will be measured with the 2-item Generalized Anxiety Disorder scale (GAD-2), which has demonstrated high sensitivity and specificity in detecting generalized anxiety disorder in primary care populations [61,62].

Pain-related activity interference with daily activities will be assessed using three items from the Graded Chronic Pain Scale (GCPS). The GCPS has been validated and shown to have good psychometric properties in a large population survey and in large samples of primary care patients with pain [63,64]. Participants will be asked to rate the following three items on a 0 to 10 scale: their current back pain (back pain “right now”), their worst back pain in the previous month and their average pain level over the previous month.

Global improvement with treatment will be measured with the Patient Global Impression of Change scale [65]. This single question asks participants to rate their improvement with treatment on a 7-point scale that ranges from “very much improved” to “very much worse,” with “no change” used as the midpoint. Global ratings of improvement with treatment provide a measure of overall clinical benefit from treatment and are considered one of the core outcome domains in pain clinical trials [49].

Use of medications and exercise for back pain during the previous week will be assessed with the 8-, 26- and 52-week questionnaires.

General health status will be assessed with the 12-item Short Form Health Survey (SF-12) [66], a widely used instrument that yields summary scores for physical and mental health status. The SF-12 will also be used to calculate quality-adjusted life-years (QALYs) using the Short Form Health Survey in 6 dimensions in the cost-effectiveness analyses [67].

Qualitative outcomes will be measured with open-ended questions. We have included open-ended questions in our previous trials and found that they yield valuable insights into participants’ feelings about the value of specific components of the interventions and the impact of the interventions on their lives. We therefore will include open-ended questions about these issues at the end of the 8-, 26- and 52-week follow-up interviews.

#### Measures used in mediator analyses

In the MBSR arm, we will evaluate the mediating effects of increased mindfulness (measured with the Nonreactivity, Observing, Acting with Awareness, and Nonjudging subscales of the Five Facet Mindfulness Questionnaire short form [68-70]) and increased pain acceptance (measured with the Chronic Pain Acceptance Questionnaire [71,72]) on the primary outcomes. In the CBT arm, we will evaluate the mediating effects of improvements in pain beliefs and/or appraisals (measured with the Patient Self-Efficacy Questionnaire [73]; the Survey of Pain Attitudes 2-item Control, Disability, and Harm scales [74-76]; and the Pain Catastrophizing Scale [77-80]) and changes in the use of pain coping strategies (measured with the Chronic Pain Coping Inventory 2-item Relaxation scale and the complete Activity Pacing scale [81,82]) on the primary outcomes. Although we expect the effects of MBSR and CBT on outcomes to be mediated by different variables, we will explore the effects of all potential mediators on outcomes in both treatment groups.

#### Measures used in the cost-effectiveness analyses

Direct costs will be estimated using cost data extracted from the electronic medical records for back-related services provided or paid by GHC and from patient reports of care not covered by GHC. Indirect costs will be estimated using the Work Productivity and Activity Impairment questionnaire [83]. The effectiveness of the intervention will be derived from the SF-12 general health status measure [84].

### Data collection, quality control and confidentiality

Data will be collected from participants by trained telephone interviewers using a computer-assisted telephone interview (CATI) version of the questionnaires to minimize errors and missing data. Questions about experiences with specific aspects of the interventions (for example, yoga, meditation, instruction in coping strategies) that would unmask interviewers to treatment groups will be asked at each time point after all other outcomes have been assessed. We will attempt to obtain outcome data from all participants in the trial, including those who never attend or drop out of the classes, those who discontinue enrollment in the health plan and those who move away. Participants who do not respond to repeated attempts to obtain follow-up data by telephone will be mailed a questionnaire including only the two primary outcome measures and offered $10 for responding.

We are will collect information at every stage of recruitment, randomization and treatment so that we can report patient flow according to the CONSORT (Consolidated Standards of Reporting Trials) guidelines [85]. To maintain the confidentiality of patient-related information in the database, unique participant study numbers will be used to identify patient outcomes and treatment data. Study procedures are in place to ensure that all masked personnel will remain masked to treatment group.

### Protection of human participants and assessment of safety

#### Protection of human participants

The GHC Institutional Review Board (IRB) approved this study.

#### Safety monitoring

This trial will be monitored for safety by an independent Data and Safety Monitoring Board (DSMB) composed of a primary care physician experienced in mindfulness, a biostatistician and a clinical psychologist with experience in treating patients with chronic pain.

#### Adverse experiences

We will collect data on adverse experiences (AEs) from several sources: (1) reports from the CBT and MBSR instructors of any participants’ experiences of concern to them; (2) the 8-, 26- and 52-week CATI follow-up interviews in which the participants are asked about any harm they felt during the CBT or MBSR treatment and any serious health problems they had had during the respective time periods; and (3) spontaneous reports from participants. The project coinvestigators and a GHC primary care internist will review AE reports from all sources weekly. Any serious AEs will be reported promptly to the GHC IRB and the DSMB. AEs that are not serious will be recorded and included in regular DSMB reports. Any identified deaths of participants will be reported to the DSMB chair within 7 days of discovery, regardless of attribution.

#### Stopping rules

The trial will be stopped only if the DSMB believes that there is an unacceptable risk of serious AEs in one or more of the treatment arms. In this case, the DSMB can decide to terminate one of the arms of the trial or the entire trial.

### Statistical issues

#### Sample size and detectable differences

Our sample size was chosen to ensure adequate power to detect a statistically significant difference between each of the two mind–body treatment groups and the usual care group, as well as power to detect a statistically significant difference between the two mind–body treatment groups. Because we considered patient activity limitations to be the more consequential of our two co–primary outcome measures, we based our sample size calculations on the modified RDQ [30]. We specified our sample size on the basis of the expected percentage of patients with a clinically meaningful improvement measured with the RDQ at the 26-week assessment (that is, at least 30% relative to baseline) [57].

Because of multiple comparisons, we will use Fisher’s protected least significant difference test [86], first analyzing if there is any significant difference among all three groups (using the omnibus *χ*^2^ likelihood ratio test) for each outcome and each time point. If we find a difference, we will then test for pairwise differences between groups. We will need 264 participants (88 in each group) to achieve 90% power to find either mind–body treatment different from usual care on the RDQ. This assumes that 30% of the usual care group and 55% of each mind–body treatment group will have clinically meaningful improvement on the RDQ at 26 weeks, rates of improvement that are similar to those we observed in a similar back pain population in an evaluation of complementary and alternative treatments for back pain [87]. We will have at least 80% power to detect a significant difference between MBSR and CBT on the RDQ if MBSR is at least 20 percentage points more effective than CBT (that is, 75% of the MBSR group versus 55% of the CBT group).

Our other co–primary outcome is the pain bothersomeness rating. With a total sample size of 264 participants, we will have 80% power to detect a difference between a mind–body treatment group and usual care on the bothersomeness rating scale, assuming that 47.5% of usual care and 69.3% of each mind–body treatment group have 30% or more improvement from baseline on the pain bothersomeness rating scale. We will have at least 80% power to detect a significant difference between MBSR and CBT on the bothersomeness rating scale if MBSR is at least 16.7 percentage points more effective than CBT (that is, 87% of the MBSR group versus 69.3% of the CBT group).

When analyzing the primary outcomes as continuous measures, we will have 90% power to detect a 2.4-point difference between usual care and either mind–body treatment on the modified RDQ scale scores and a 1.1-point difference between usual care and either mind–body treatment on the pain bothersomeness rating scale (assumes normal approximation to compare two independent means with equal variances and a two-sided *P* = 0.05 significance level with standard deviations of 5.2 and 2.4 for RDQ and pain bothersomeness measures, respectively [88]. Assuming an 11% loss to follow-up (slightly higher than that found in our previous back pain trials), we plan to recruit a sample of 297 participants (99 per group).

Both of the co–primary outcomes will be tested at the *P* < 0.05 level at each time point because they address separate scientific questions. Analyses of both outcomes at all follow-up time points will be reported, imposing a more stringent requirement than simply reporting a sole significant outcome.

### Statistical analyses

#### Primary analyses

In our comparisons of treatments based on the outcome measures, we will analyze outcomes assessed at all follow-up time points in a single model, adjusting for possible correlation within individuals and treatment group cohorts using generalized estimating equations [89]. Because we cannot reasonably make an assumption regarding constant or linear group differences over time, we will include an interaction term between treatment groups and time points. We plan to adjust for baseline outcome values, sex and age, as well as other baseline characteristics found to differ significantly by treatment group or follow-up outcomes, to improve precision and power of our statistical tests. We will conduct the following set of analyses for both the continuous outcome score and the binary outcome (clinically significant change from baseline), including all follow-up time points (4, 8, 26 and 52 weeks). The MBSR treatment will be deemed successful only if the 26-week time point comparisons are significant. The other time points will be considered secondary evaluations.

We will use an intent-to-treat approach in all analyses; that is, the assessment of individuals will be analyzed by randomized group, regardless of participation in any classes. This analysis minimizes biases that often occur when participants who do not receive the assigned treatments are excluded from analysis. The regression model will be in the following general form:

$$\begin{array}{ll} g\left(y_{t}\right)= & \beta_{0}+\beta_{1}\left(\mathrm{baseline}\right)+\alpha_{1}\left(\mathrm{treatment}\right)+\alpha_{2}\left(\mathrm{time}\right)\\ & +\alpha_{3}\left(\mathrm{treatment}\right)\times \mathrm{time}+\alpha_{4}z+\epsilon , \end{array}$$

where *y*_t_ is the response at follow-up time *t*, *baseline* is the prerandomization value of the outcome measure, *treatment* includes dummy variables for the MBSR and CBT groups, *time* is a series of dummy variables indicating the follow-up times and *z* is a vector of covariates representing other variables adjusted for. (Note that α_1_, α_2_, α_3_ and α_4_ are vectors.) The referent group in this model is the usual care group. For binary and continuous outcomes, we will use appropriate link functions (for example, logit for binary). For each follow-up time point at which the omnibus *χ*^2^ test is statistically significant, we will go on to test whether there is a difference between MBSR and usual care to address aim 1 and a difference between MBSR and CBT to address aim 2. We will also report the comparison of CBT to usual care. When determining whether MBSR is an effective treatment for back pain, we will require that aim 1, the comparison of MBSR to usual care, must be observed.

On the basis of our previous back pain trials, we expect at least an 89% follow-up and, if that holds true, our primary analysis will be a complete case analysis, including all observed follow-up outcomes. However, we will adjust for all baseline covariates that are predictive of outcome, their probability of being missing and differences between treatment groups. By adjusting for these baseline covariates, we assume that the missing outcome data in our model are missing at random (given that baseline data are predictive of missing data patterns) instead of missing completely at random. We will also conduct sensitivity analysis using an imputation method for nonignorable nonresponses to evaluate whether our results are robust enough to compensate for different missing data assumptions [90].

##### *Mediator analyses*

If MBSR or CBT is found to be effective (relative to usual care and/or to each other) in improving either primary outcome at 26 or 52 weeks, we will move to aim 3 to identify the mediators of the effects of MBSR and group CBT on the RDQ and pain bothersomeness scale. We will perform the series of mediation analyses separately for the two primary outcomes (RDQ and pain bothersomeness scale scores) and for each separate treatment comparator of interest (usual care versus CBT, usual care versus MBSR and CBT versus MBSR). We will conduct separate mediator analyses for the 26- and 52-week outcomes (if MBSR or CBT is found to be effective at those time points).

Next, we describe in detail the mediator analysis for the 26-week time point. A similar analysis will be conducted for the 52-week time point. We will apply the framework of the widely used approach of Baron and Kenny [91]. Once we have demonstrated the association between the treatment and the outcome variable (the “total effect” of the treatment on the outcome), the second step will be to demonstrate the association between the treatment and each putative mediator. We will construct a regression model for each mediator with the 4- or 8-week score of the mediator as the dependent variable and the baseline score of the mediator and treatment indicator as independent variables. We will conduct this analysis for each potential mediator and will include as potential mediators in the following step only those that have a P-value ≤0.10 for the relationship with the treatment. The third step will be to demonstrate the reduction of the treatment effect on the outcome after removing the effect of the mediators. We will construct a multimediator inverse probability weighted (IPW) regression model [92]. This approach will allow us to estimate the direct effects of treatment after rebalancing the treatment groups with respect to the mediators. Specifically, we will first model the probability of the treatment effects, given the mediators (that is, all mediators that were found to be associated with treatment in step 2), using logistic regression and adjusting for potential baseline confounders. Using this model, we will obtain the estimated probability that each person received the observed treatment, given the observed mediator value. We will then use an IPW regression analysis to model the primary outcomes on treatment status while adjusting for the baseline levels of the outcome and mediator. Comparing the weighted model with the unweighted model will allow us to estimate how much of the direct effect of treatment on the associated outcome can be explained by each potential mediator. The inclusion in step 3 of all mediators found to be significant in step 2 will enable us to examine whether the specific variables that we hypothesized would differentially mediate the effects of MBSR versus CBT in fact mediate the effects of each treatment independently of the effects of the other “process variables”.

#### Cost-effectiveness analyses

A societal perspective cost–utility analysis (CUA) will be performed to compare the incremental societal costs revealed for each treatment arm (direct medical costs paid by GHC and the participant plus productivity costs) to incremental effectiveness in terms of change in participants’ QALYs [93]. This analysis will be possible only for study participants recruited from GHC. This CUA can be used by policymakers concerned with the broad allocation of health-related resources [94,95]. For the payer perspective, direct medical costs (including intervention costs) will be compared to changes in QALYs. This CUA will help us to determine whether it makes economic sense for MBSR to be a reimbursed service among this population. A bootstrap methodology will be used to estimate confidence intervals [96]. In secondary analyses conducted to assess the sensitivity of the results to different cost outcome definitions, such as varying assumptions of wage rates used to value productivity and the inclusion of non-back-related health-care resource utilization [97] in the total cost amounts, will also be considered. In cost-effectiveness analyses, we will use intention to treat and adjust for health-care utilization costs in the one calendar year prior to enrollment and for baseline variables that might be associated with treatment group or outcome, such as medication use, to control for potential confounders. We expect there will be minimal missing data, but sensitivity analyses (as described above for the primary outcomes) will also be performed to assess cost measures.

## Discussion

In this trial, we will seek to determine whether an increasingly popular approach for dealing with stress—mindfulness-based stress reduction—is an effective and cost-effective treatment option for persons with chronic back pain. Because of its focus on the mind as well as the body, MBSR has the potential to address some of the psychosocial factors that are important predictors of poor outcomes. In this trial, we will compare the effectiveness and cost-effectiveness of MBSR with that of CBT, which has been found to be effective for back pain but is not widely available. The study will also explore psychosocial variables that may mediate the effects of MBSR and CBT on patient outcomes. If MBSR is found to be an effective and cost-effective treatment option for persons with chronic back pain, it will be a valuable addition to the treatment options available for patients with significant psychosocial contributors to this problem.

## Trial status

Recruitment started in August 2012 and was completed in April 2014.

## Abbreviations

AE: Adverse event; CAM: Complementary and alternative medicine; CATI: Computer-assisted telephone interview; CBT: Cognitive-behavioral therapy; CLBP: Chronic low back pain; CUA: Cost–utility analysis; DSMB: Data and Safety Monitoring Board; GHC: Group Health Cooperative; ICD-9: International *Classification of Diseases Ninth Revision*; IPW: Inverse probability weighting; IRB: Institutional Review Board; MBSR: Mindfulness-based stress reduction; NCCAM: National Center for Complementary and Alternative Medicine; QALY: Quality-adjusted life-year.

## Competing interests

The authors declare that they have no competing interests.

## Authors’ contributions

DC and KS conceived of the trial. DC, KS, BB, JT, AC, BS, PH, RD and RH participated in refining the study design and implementation logistics and in the selection of outcome measures. AC developed plans for the statistical analyses. JT and AC developed plans for the mediator analyses. BS, BB and JT developed the materials for the CBT intervention. PH developed plans for the cost-effectiveness analyses. DC drafted the manuscript. All authors participated in the writing of the manuscript and read and approved the final manuscript.
